# Low-grade papillary Schneiderian carcinoma with *TP53* mutation: a case report and review of the literature

**DOI:** 10.1186/s13000-023-01334-8

**Published:** 2023-04-11

**Authors:** Sayaka Yuzawa, Tomohiko Michizuka, Rika Kakisaka, Yusuke Ono, Manami Hayashi, Miki Takahara, Akihiro Katada, Yusuke Mizukami, Mishie Tanino

**Affiliations:** 1 Department of Diagnostic Pathology, Asahikawa Medical University Hospital, 2-1-1-1, Midorigaoka Higashi, Asahikawa, Hokkaido 078-8510 Japan; 2 Department of Otolaryngology Head and Neck Surgery, Asahikawa Medical University, Asahikawa, Hokkaido Japan; 3 Institute of Biomedical Research, Sapporo Higashi Tokushukai Hospital, Sapporo, Hokkaido Japan; 4 Department of Medicine, Asahikawa Medical University, Asahikawa, Hokkaido Japan

**Keywords:** Low-grade papillary Schneiderian carcinoma, Sinonasal tract, *TP53*, *DEK::AFF2* fusion

## Abstract

**Background:**

Low-grade papillary Schneiderian carcinoma (LGPSC) is a relatively new entity of the sinonasal tract and is characterized by a bland morphology simulating sinonasal papilloma, invasive growth pattern with pushing borders, and aggressive clinical behavior with multiple recurrences and metastatic potential. Recently, *DEK::AFF2* fusions were identified in LGPSC. However, some LPGSCs lack *DEK::AFF2* fusion, and the molecular features of these tumors have not been clarified.

**Case presentation:**

A 69-year-old man presented with a discharge of pus from his left cheek. Computed tomography revealed a mass involving the left maxillary sinus, ethmoid sinus, and nasal cavity with the destruction of the orbital wall. The biopsy specimens showed that the tumor had a predominantly exophytic, papillary growth and did not have an apparent stromal invasion. The tumor was composed of multilayered epithelium that showed bland morphology with a round to polygonal shape, abundant eosinophilic cytoplasm, and uniform nuclei. Dense neutrophilic infiltrates were focally present. Immunohistochemically, CK5/6 was strongly and diffusely positive, and p16 was negative. p63 was mainly positive in the basal layer, and EMA was predominantly expressed in the outermost cell layer. DNA-based targeted sequencing showed *TP53* R175H mutation, whereas neither *EGFR* nor *KRAS* mutation was identified. Reverse transcription polymerase chain reaction and fluorescence in situ hybridization revealed no *DEK::AFF2* fusion.

**Conclusions:**

We describe the first case of *TP53*-mutant LGPSC and review the literature. LGPSC is a genetically heterogeneous entity, and the recognition of this rare entity and comprehensive assessment of clinicopathological and molecular findings are crucial for the correct pathological diagnosis and clinical management.

## Background

Low-grade papillary Schneiderian carcinoma (LGPSC) was first described by Lewis et al*.* in 2015, as a bland, benign-looking papillary carcinoma, which in almost all aspects resembled a sinonasal (Schneiderian) papilloma clinically and pathologically but which recurred locally and metastasized to lymph nodes, resulting in the death of the patient [1]. Since its first description, only 17 cases have been described in the literature so far [1–10]. Histologically, the tumors show predominantly exophytic and inverted papillary lesions and lack malignant cytological features. Tumor epithelia are multilayered and arranged in an orderly pattern without cilia. Most previous reports indicated that the original diagnoses were benign sinonasal tumors, including exophytic and inverted papilloma. However, during the clinical course, the tumors extended into the adjacent structures, such as the nasopharynx, middle ear, temporal bone, cheek soft tissue, and orbit [6]; the diagnoses were subsequently revised to LGPSC. In terms of pathogenesis, LGPSC lacks either virus infection or activating mutations of the MAPK pathway, which are common in sinonasal carcinoma [11] and carcinoma associated with sinonasal papilloma [12], respectively. In 2021, *DEK::AFF2* fusions were identified in LGPSC [8]. However, some LPGSCs lack *DEK::AFF2* fusion, and the molecular features of these tumors have not been clarified.

Here, we describe a case of LGPSC which involved the sinonasal region and invaded to the skin and orbital wall. Subsequent molecular analysis revealed *TP53* missense mutation, but *DEK::AFF2* fusion, *EGFR,* and *KRAS* mutation were not identified. Invasive growth by clinical and radiological findings and distinctive morphology with p53 immunopositivity provided diagnostic clues for this rare disease. Recognizing this rare entity is important for the correct pathological diagnosis and appropriate clinical management.

### Case presentation

**Fig. 1:**
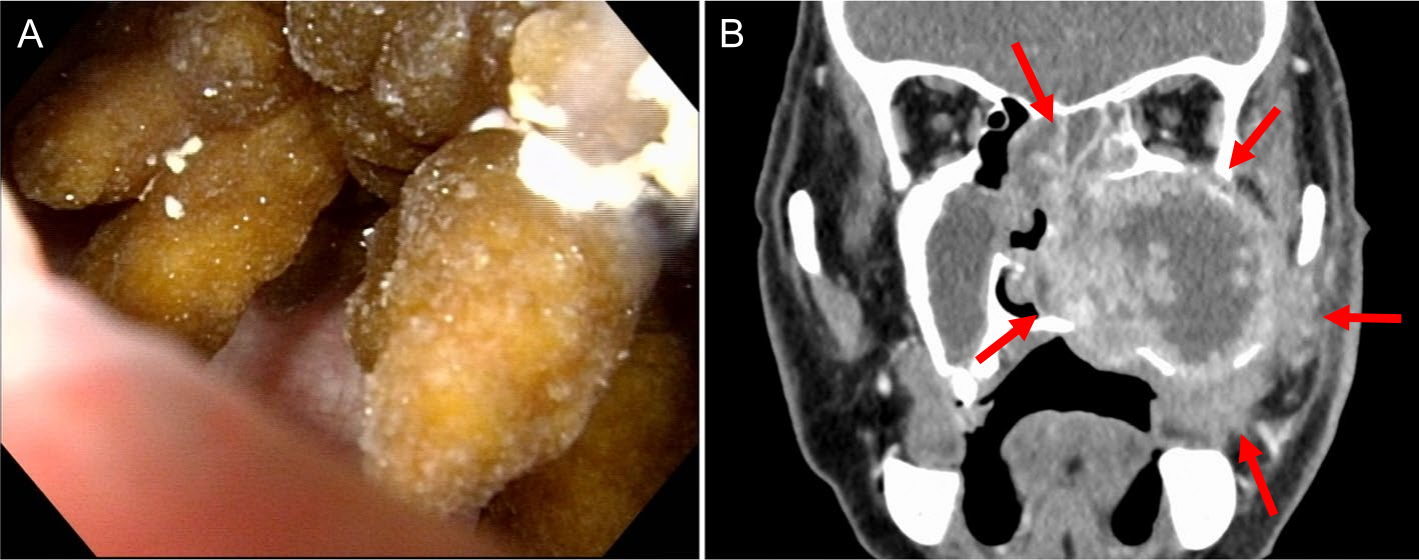
Clinical and radiographic presentation. **A** A brown to yellowish papillary tumor was noted in the left maxillary and ethmoid sinuses on nasal endoscopy. **B** Computed tomography revealed a tumor of left maxillary sinus extending to left nasal cavity, ethmoid sinus, and orbital floor

A 69-year-old man presented with swelling of his left cheek and discharge of pus from the skin of his cheek. He also a had persistent nasal obstruction and purulent rhinorrhea. A papillary mass was noted in the left maxillary and ethmoid sinuses on nasal endoscopy (Fig. 1A). The mass protruded to the oral cavity through the hard palate. Computed tomography and magnetic resonance imaging revealed a tumor of the left maxillary sinus extending to the left nasal cavity, ethmoid sinus, and orbital floor, measuring about 90 × 50 mm (Fig. 1B). Biopsy was performed from the maxillary sinus.

**Fig. 2:**
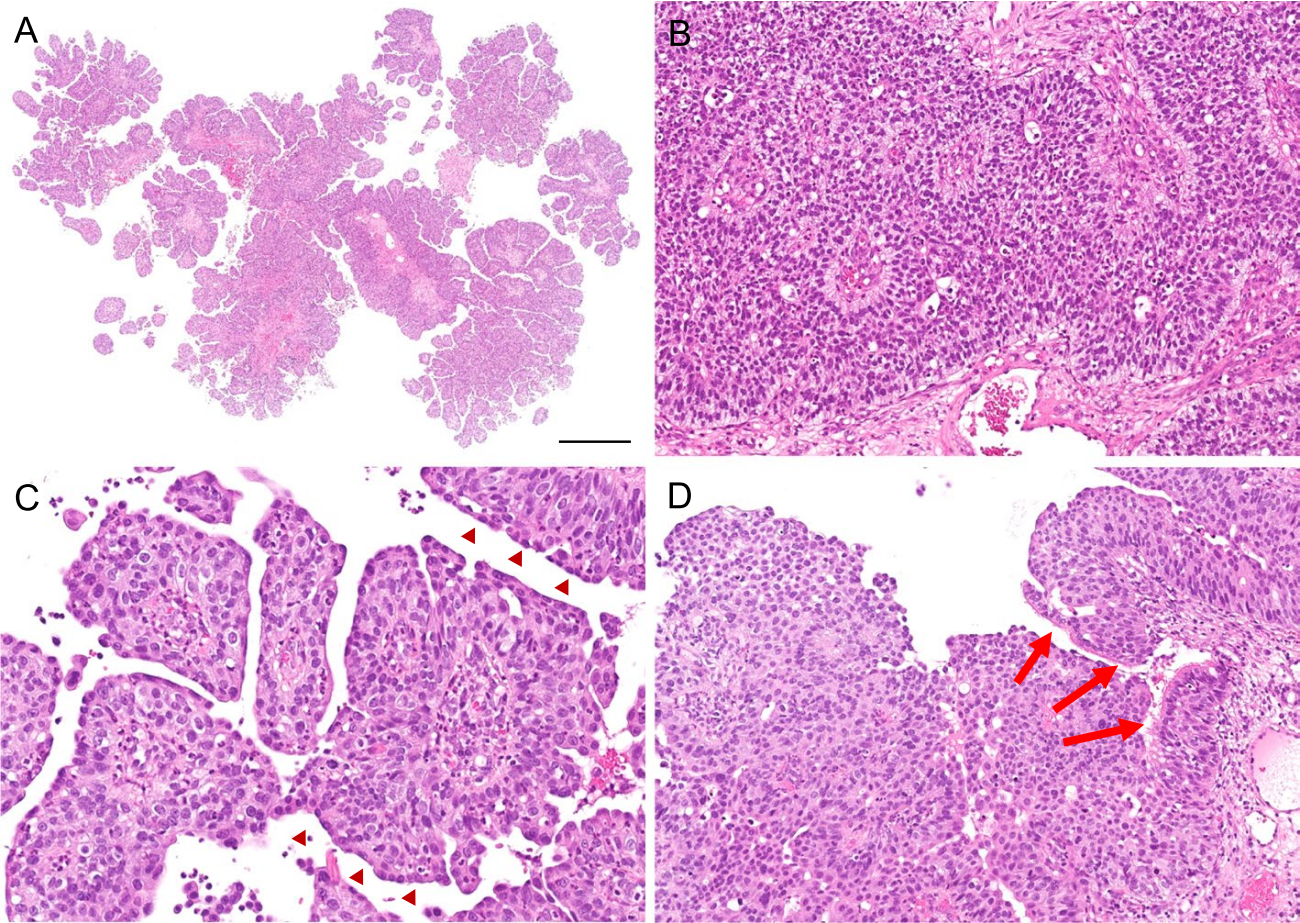
Microscopic findings. **A** The tumor was composed of exophytic, papillary growth of multilayered epithelium. Scale bar 1 mm. **B** A palisading pattern of columnar cells with reverse polarity was observed in the basal layer. **C** The tumor cells were composed of uniformly round and polygonal cells with abundant, eosinophilic cytoplasm and monomorphic round nuclei with fine chromatin. Discontinuous flattened cells were found in the outermost layer of multilayered epithelium (arrowheads). **D** Focal ciliated epithelium from residual respiratory mucosa was found (arrows) while no dysplasia-carcinoma sequence was observed

Microscopically, the tumor was composed of an exophytic, papillary growth of multilayered epithelium (Fig. 2A). Some areas showed inverted, anastomosing ribbons with pushing borders. A palisading pattern of columnar cells with reverse polarity was observed in the basal layer (Fig. 2B). The tumor cells were composed of uniformly round and polygonal cells with abundant, eosinophilic cytoplasm and monomorphic round nuclei with fine chromatin and occasional small nucleoli. Discontinuous flattened cells were found in the outermost layer of multilayered epithelium (Fig. 2C). Neutrophilic infiltrates were focally present in both tumor nests and stroma. A focal peculiar acantholytic change was seen. A few scattered mucous cells were found in the middle layer. There was focal ciliated epithelium from residual respiratory mucosa (Fig. 2D), while no dysplasia-carcinoma sequence was observed. There was no overt stromal invasion, lymphovascular invasion, or perineural invasion. Keratinization and necrosis were not found through the lesion. The mitotic rate was relatively low, although increased mitotic activity (up to 38/10 high-power fields) was observed focally.

**Fig. 3:**
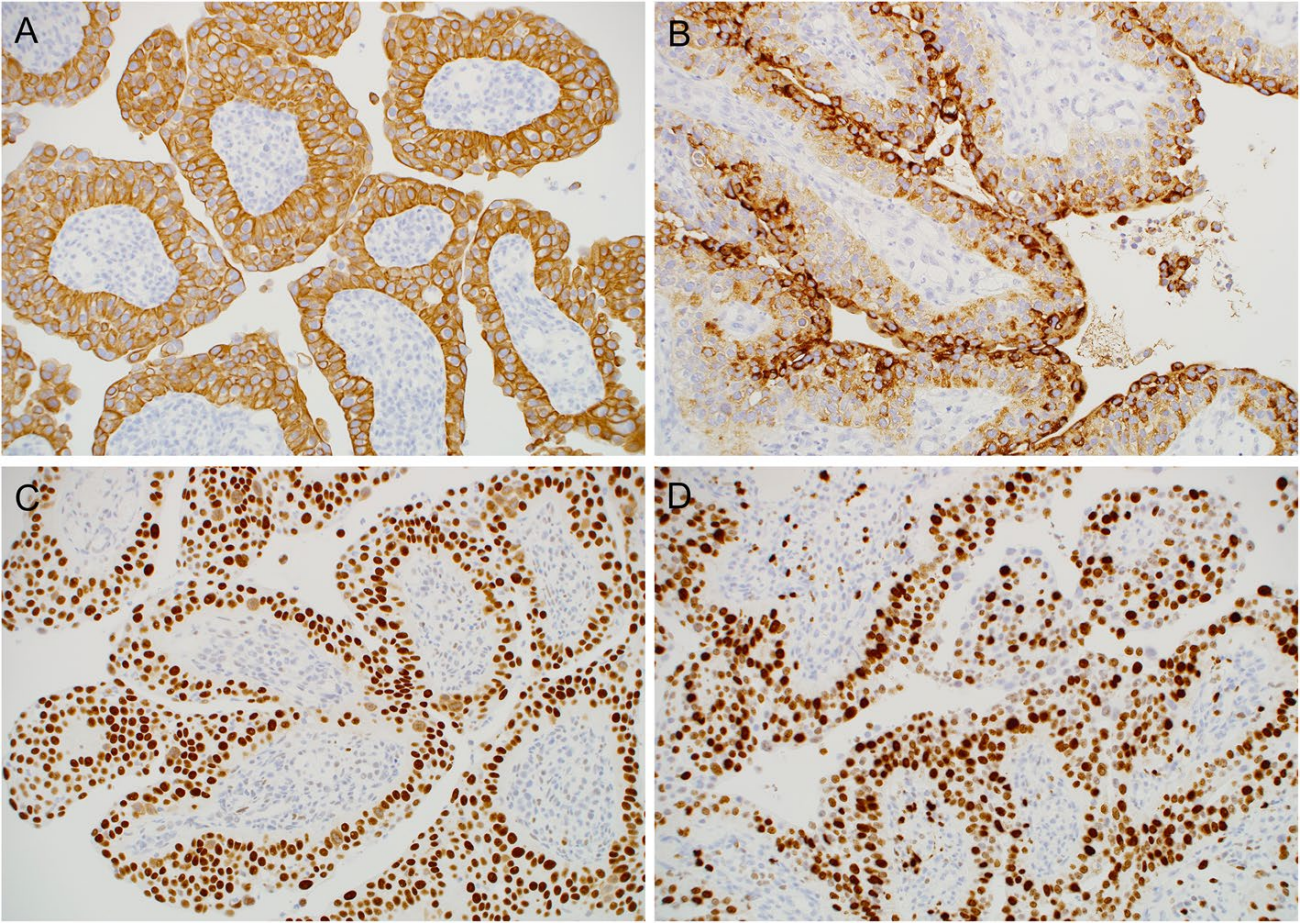
Immunohistochemical findings. **A** The tumor was diffusely and strongly positive for CK5/6. **B** EMA was mainly expressed in the outermost cell layer. **C** p53 was strongly positive in almost all tumor cell nuclei. **D** The Ki67 labeling index was about 90%

By immunohistochemistry, the tumor was diffusely and strongly positive for CK5/6 (Fig. 3A) and high molecular weight cytokeratin. p63 was mainly positive in the basal layer, and EMA was predominantly expressed in the outermost cell layer (Fig. 3B). CK7 and p16 were negative in tumor cells. p53 was strongly positive in almost all tumor cells (Fig. 3C). The Ki67 labeling index was about 90% (Fig. 3D). Based on the radiological and pathological findings, a diagnosis of LGPSC was rendered.

**Fig. 4:**
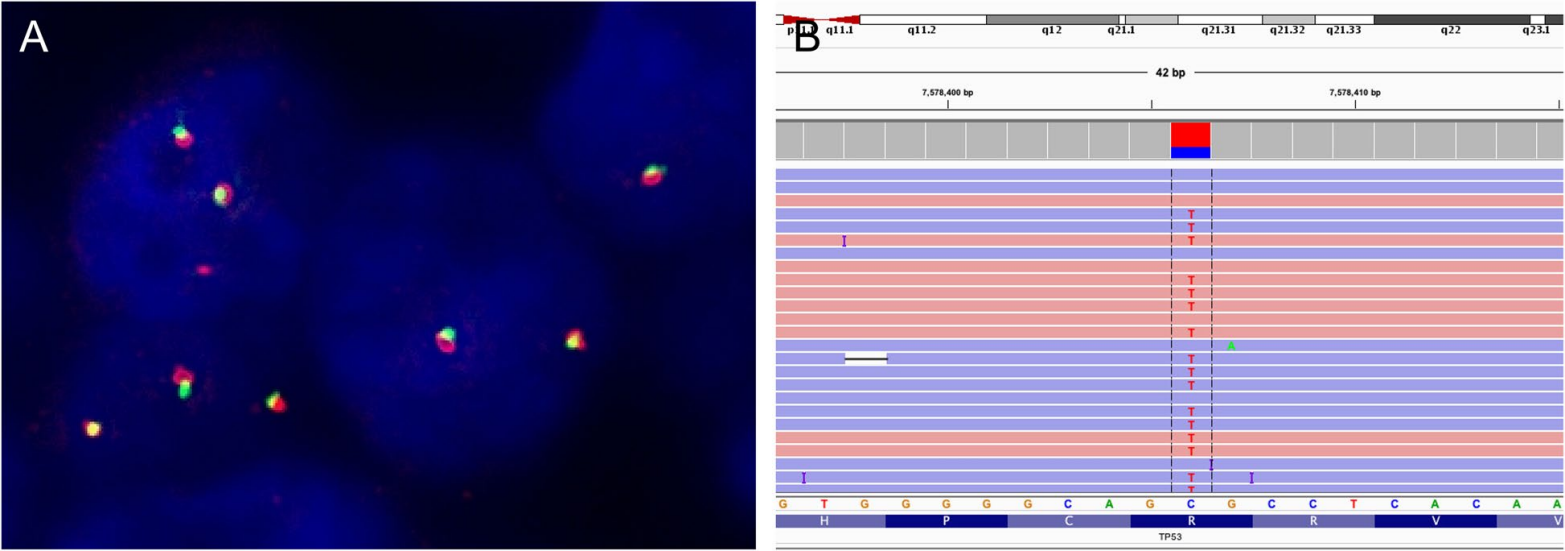
FISH and DNA-based targeted sequencing. **A** FISH using a *DEK* break-apart probe revealed no *DEK* rearrangement. **B** IGV alignment of the *TP53* R175H (c.524G > A)

*DEK::AFF2* fusion was analyzed by reverse transcription polymerase chain reaction (RT-PCR) as described previously [8] and confirmed the absence of *DEK::AFF2* fusion transcripts (data not shown). Fluorescence in situ hybridization (FISH) using a *DEK* break-apart FISH probe (CytoTest, Rockville, MD, USA) revealed no *DEK::AFF2* fusion (Fig. 4). DNA-based targeted sequencing was performed using Ion AmpliSeq™ Cancer Hotspot Panel v2 (Thermo Fisher Scientific, Waltham, MA), targeting mutation hotspots of 50 cancer-related genes. The sequencing confirmed *TP53* R175H mutation (69.1% variant allele frequency, with sequencing depth ~ 1000 ×). There were no hotspot mutations in *EGFR*, *KRAS*, and *CDKN2A* genes.

Because the primary tumor was not surgically resectable, he underwent radiotherapy with 66 Gy in 33 fractions and concomitant intra-arterial chemotherapy. Twelve months after the initiation of treatment, no residual tumor was observed by positron emission tomography.

## Discussion and conclusions

LGPSC is a unique type of sinonasal carcinoma that is considerably distinct from conventional sinonasal papilloma and other carcinomas in the sinonasal region and shows a propensity for multiple recurrences, lymph node metastasis, and ultimate mortality [1]. LGPSC is a relatively new entity, and only eighteen cases of LGPSC were clinicopathologically described in the literature, including our case [1–10]. In the current WHO Classification of Tumours (5th ed.) [13], LGPSC is newly cited in the section of non-keratinizing squamous cell carcinoma of the nasal cavity, paranasal sinuses, and skull base. *DEK::AFF2* squamous carcinoma, the newly described subtype of non-keratinizing squamous cell carcinoma in the current WHO classification shows substantial morphologic overlap with tumors reported as LGPSC. Recent data suggest that some LGPSCs also harbor *DEK::AFF2* fusions; however, the genetic alterations other than *DEK::AFF2* fusions remain unknown. We examined genetic changes in the case of LGPSC using DNA-based targeted sequencing, RT-PCR, and FISH and discovered the new genetic change of *TP53* R175H but no *DEK::AFF2* fusion.

The clinicopathological and molecular findings of previously reported cases of LGPSC and our case are summarized in Table 1. Briefly, 13 patients were female, 4 were male, and one was unknown, with a median age of 56 years (range 18–82 years). Although nasal obstruction is common, the presenting symptoms of LGPSC are variable and non-specific, depending on the involved sites of the tumor. Some patients experienced facial pain, sensory or auditory neuropathies, loss of swelling, tinnitus, or significant facial swelling [3, 4, 6, 7], indicating the infiltrative nature of this aggressive tumor. As shown here, most tumors were initially diagnosed as benign neoplasms, such as inverted papilloma and oncocytic papilloma. However, the revision of the original samples documented peculiar histopathological features distinct from conventional sinonasal papillomas. Four of the 18 cases developed nodal metastases, one distant metastasis, and two died of progressive disease. Although the Ki67 labeling index was very high in our case, other clinicopathological features were similar to those of other LGPSCs.

**Table 1:** Cases of previously reported low-grade papillary Schneiderian carcinoma

No	Age	Sex	Initial symptoms	Original diagnosis	Involved site	Local recurrence	Nodal metastasis	Distant metastasis	Adjunctive treatment	Follow-up	p53	Ki67	HPV	*EGFR, KRAS*	*DEK-AFF2*	Reference
1	47	F	Nasal obstruction, polyps	Fungiform Schneiderian papilloma	ES, NC, MS, FS, cheek, OB	> 10 times (18 years)	Yes	No	CT, RT	18 years, DOD	50%	5%	-	WT	exon7-exon4	[1, 7, 8]
2	42	F	Nasal obstruction, rhinorrhea	Oncocytic papilloma	NC, MS, ES	Once (3 years)	No	No	RT	21 months, NED	50%	10%	-	NA	NA	[2]
3	65	F	Nasal obstruction	Schneiderian papilloma	NC, MS, ME, ET	Once (1 year)	No	No	RT	3 months, NED	NA	NA	NA	NA	NA	[3]
4	56	F	Nasal discharge, facial pain	Transitional proliferation with inverted growth	MS, ES, NC, PF, OB, IF	Once (1 year)	No	No	No	> 12 months, NED	Positive	< 10%	-	NA	NA	[4]
5	68	M	Nasal obstruction	Low-grade papillary Schneiderian carcinoma	NC	No	No	No	No	16 months, NED	50%	2–50%	-	WT	NA	[5]
6	53	F	Nasal obstruction, loss of smell, headache	Sinonasal papilloma	NC, NP, ME, ET	Four times	No	No	RT	48 months, AWD	20%	5%	-	WT	NA	[6]
7	64	F	Nasal obstruction, epistaxis, loss of smell	Inverted papilloma	NC, FS, MS, ES, SS, IF, PF, ME, NP	Four times	No	No	No	7 months, DOD	10%	5%	-	WT	NA	[6]
8	18	F	Nasal obstruction, epistaxis	Papilloma	NC, ES, MS, ET, ME, NP	Nine times	Yes	lung	CT, RT	13 months, AWD	20%	5%	-	WT	NA	[6]
9	53	F	Nasal obstruction, epistaxis, epiphora tinnitus, aural fullness	Papilloma	NC, SS, SB, ET, ME, CS, NP	Twice	Yes	No	RT	18 months, AWD	30%	10%	-	WT	exon7-exon5	[6, 8]
10	51	F	Nasal obstruction, purulent nasal discharge, epistaxis	Exophytic papilloma	NC	Twice	No	No	RT	30 months, NED	10%	5%	-	WT	exon7-exon5	[6, 8]
11	NA	NA	(from No. 11 to 14) Neurological symptoms (sensory or auditory neuropathies), facial swelling, nasal obstruction and drainage	(from No. 11 to 14) Inverted papilloma, atypical squamous proliferation with papillary features, low-grade nonkeratinizing SCC, SCC in situ arising in an inverted papilloma	NC	No	No	No	CT, RT	NED	(from No. 11 to 14) average 43% (15–70%)	(from No. 11 to 14) average 27% (5–50%)	-	WT	NA	[7]
12	NA	NA			MS	No	No	No	CT, RT	NED			-	WT	NA	[7]
13	NA	NA			Mastoid air cells, ME	No	No	No	No	NED			-	WT	NA	[7]
14	NA	NA			NC	Once (10 months)	No	No	No	15 months, AWD			-	WT	NA	[7]
15	NA	NA	NA	NA	NA	NA	NA	NA	NA	NA	NA	NA	-	WT	+	[8]
16	64	F	NA	LGPSC	NC, ES	NA	No	No	NA	NA	NA	NA	-	NA	+	[9]
17	76	F	Asymptomatic (detected by PET)	Inverted papilloma or LGPSC	NP	No	Yes	No	RT	13 months, DOA	NA	15%	-	NA	NA	[10]
18	69	M	Nasal obstruction, cheek swelling, purulent rhinorrhea	LGPSC	MN, ES, NC, OB, Cheek	Not resectable	No	No	CT, RT	8 months, AWD	100%	90%	-	WT	-	This case

p53 and Ki67 are scores at the primary tumor of initial surgery/biopsy. HPV was tested by PCR, in situ hybridization, or p16 immunohistochemistry

*AWD* Alive with disease, *CIS* Carcinoma in situ, *CT* Chemotherapy, *CS* cavernous sinus, *DOA* Died of another disease, *DOD* Died of disease, *ES* Ethmoid sinus, *ET* Eustachian tube, *F* Female, *FS* Frontal sinus, *IF* Infratemporal fossa, *M* Male, *ME* Middle ear, *MS* Maxillary sinus, *NA* not available, *NC* Nasal cavity, *NED* No evidence of disease, *NP* Nasopharynx, *OB* Orbit, *PET* Positron emission tomography, *PF* Pterygopalatine fossa, *RT* Radiation therapy, *SB* Skull base, *SCC* Squamous cell carcinoma, *SS* Sphenoid sinus, *WT* Wild type

Thirteen out of the 18 cases of LGPSC were analyzed genetically and revealed no *EGFR* and *KRAS* hotspot mutations, which are known as the driver mutations in inverted and oncocytic sinonasal papillomas, respectively, and their associated carcinomas. This indicates that LGPSC is not associated with benign sinonasal papillomas genetically, despite the morphologic resemblance.

*DEK::AFF2* fusion was first reported in the literature in a patient with skull base squamous cell carcinoma who was an exceptional responder to programmed cell death protein 1 inhibitor therapy [14]. Since this case report, about 30 cases of sinonasal carcinoma with *DEK::AFF2* fusion were described in the literature using RNA sequencing, FISH, or RT-PCR [8, 9, 15–17]. About 40% of *DEK::AFF2* carcinoma showed high-grade morphology, whereas the others were low-grade with morphological overlap with LGPSC [8, 17]. Indeed, six out of the 18 patients of LGPSC were tested for *DEK::AFF2* fusion and five patients other than our case showed *DEK::AFF2* fusion (Cases 1, 9, 10, 15, and 16). More recently, Kuo et al. described that 68.6% (11/16 patients) of sinonasal tumors showing features of LGPSC had *DEK::AFF2* fusion; however, they did not show the other genetic alterations in *DEK::AFF2*-negative LGPSC [17], which indicates that LGPSC is a genetically more heterogeneous entity.

*TP53* is one of the most mutated genes in human cancers, including head and neck carcinomas. *TP53* is frequently mutated in HPV-negative head and neck squamous cell carcinomas but not in HPV-positive tumors [18]. In the LGPSCs, five cases were examined for *TP53* mutation by targeted sequencing, and none of them had *TP53* mutations [7]. Moreover, none of the previously reported cases of LGPSC showed strong, diffuse expression or complete absence, suggesting missense and nonsense *TP53* mutation, respectively. Therefore, our case is the first case of LGPSC with *TP53* mutation.p53 immunohistochemistry is the most sensitive marker for *TP53* mutation, and diffuse expression of p53 was observed in our case. In cases without *TP53* mutations, LGPSC showed *TP53* positivity in 10–50% of tumor cells (Table 1), which is generally higher than those of sinonasal papilloma. Therefore, p53 immunohistochemistry is very useful for the differential diagnosis of sinonasal papillary tumors.

The *TP53* R175 hotspot is located at the zinc-binding site near the DNA binding interface, which is essential to maintaining structural stability. The p53-R175H mutation causes global conformational changes leading to indirect disruption of p53-DNA interaction [19]. Moreover, the p53-R175H gains function by binding to some DNA sequences which are different from the wild-type p53 and transactivating its target genes [20], leading to promote cancer cell proliferation, migration, invasion, initiation, metabolic reprogramming, and angiogenesis. However, the function of p53-R173H is highly dependent on the cellular context. Therefore, the reason why *TP53* mutation causes the same histomorphology as LGPSC with *DEK::AFF2* is unclear. The limitation is that we did not perform whole-genome sequence or RNA-based sequencing, so the possibility of another novel fusion may have been missed in our case.

The differential diagnosis of LGPSC is sinonasal papilloma and carcinoma arising in a sinonasal papilloma. Sinonasal papillomas show respiratory-type columnar-to-squamous gradation with a mixture of immature squamous, ciliated, mucous, and focally more maturing squamous cells. They have no cytological atypia with only rare mitoses and lack destructive or irregular, infiltrative growth [21]. Conventional sinonasal papilloma with associated carcinoma shows prominent architectural abnormalities, moderate to severe cytologic atypia, pleomorphism, high mitotic activity, and/or necrosis [22]. *EGFR* and *KRAS* mutations were known as a driver mutation of sinonasal papilloma and carcinoma associated with sinonasal papilloma [12], and *TP53* mutations and *CKDN2A* alterations were reported to be associated with malignant transformation of sinonasal papilloma [23]. In our case, the tumor showed bland cytomorphology, abundant eosinophilic cytoplasm, and the absence of respiratory epithelial cells, keratinization, and glandular differentiation, which were histological diagnostic clues for LGPSC [6]. Although *TP53* mutation was identified, no hotspot mutations in *EGFR*, *KRAS*, and *CDKN2A* were identified. There remains a possibility of carcinoma arising in a sinonasal papilloma since only a small biopsy was submitted for histopathological examination.

In summary, we described a case of LGPSC with *TP53* mutation but without *DEK::AFF2* fusion. Mutation of *TP53* may play a crucial role in the pathogenesis of *DEK::AFF2*-negative LGPSC. LGPSC could easily be underdiagnosed as a sinonasal papilloma at initial presentation due to a deceptively bland morphology without overt stromal invasion, especially in a case of a small biopsy specimen. Given its aggressive nature, an appropriate diagnosis requires a comprehensive assessment of clinical history, radiological imaging, morphology, and ancillary testing for p53, p16/HPV, *EGFR/KRAS* mutations, and *DEK* rearrangement.

## Data Availability

The datasets used and/or analysed during the current study are available from the corresponding author on reasonable request.

## Abbreviations

LGPSC: Low-grade papillary Schneiderian carcinoma
RT-PCR: Reverse transcription polymerase chain reaction
FISH: Fluorescence in situ hybridization
